# Quantitative Analysis of Colistin-Resistant *Escherichia coli* in Retail Meat from Local Vietnamese Markets

**DOI:** 10.1155/2021/6678901

**Published:** 2021-02-19

**Authors:** Thang N. Nguyen, Diep T. Khong, Ha V. Le, Hoa T. Tran, Quang N. Phan, Huong T. T. Le, Ryuji Kawahara, Yoshimasa Yamamoto

**Affiliations:** ^1^Center of Medical-Pharmaceutical Science and Technology Services, Thai Binh University of Medicine and Pharmacy, Thai Binh, Vietnam; ^2^Department of Microbiology, Osaka Institute of Public Health, Osaka 537-0025, Japan; ^3^Life Science Research Center, Gifu University, Gifu 501-1194, Japan

## Abstract

The spread of drug-resistant bacteria via food has contributed to the dissemination of resistant bacteria among humans. However, the status of food contamination with resistant bacteria, particularly the quantitative level of resistant bacteria in food, has not yet been well elucidated. In this study, the abundance of colistin-resistant *Escherichia coli* in meat samples was quantified to understand the origin of the contamination of meat available in local Vietnamese markets. Fifteen samples each of chicken and pork meat purchased from local Vietnamese markets were assessed for the presence of colistin-resistant *E*. *coli* with the mobile colistin resistance gene, *mcr*. The results showed that 40% (6/15) and 66% (10/15) of the pork and chicken meat samples, respectively, were contaminated with colistin-resistant *E*. *coli*. The median quantitative levels of colistin-resistant *E*. *coli* in the contaminated pork and chicken samples were 1.8 × 10^4^ and 4.2 × 10^3^ CFU/g, respectively. The results of phylogenetic analysis of isolates from a chicken meat sample showed that the contaminated colistin-resistant *E*. *coli* was a mix of multiple phylogenetical clones of bacteria that may have multiplied during sale. This is the first study to quantify the abundance of colistin-resistant *E*. *coli* in meat samples.

## 1. Introduction

The antibiotic colistin is used as the last resort for treating severe infections caused by multidrug-resistant Gram-negative bacteria, particularly carbapenem-resistant bacteria [1]. The recent emergence of acquired colistin resistance is considered a threat to global public health. Following the discovery of the first mobile colistin resistance gene, *mcr-1*, in 2015 [2], other *mcr* variants (*mcr-2* to *mcr-10*) were also detected [3]. Thus, colistin resistance has disseminated worldwide, necessitating studies for monitoring and controlling the emergence of bacteria resistant to multiple drugs, including colistin.

Colistin has been widely used in animal husbandry, mostly orally, for various purposes [4–6]. In livestock, colistin may contribute to the emergence of colistin-resistant (CR) bacteria due to selective pressure [4, 7]. Studies from Vietnam have shown that colistin and/or colistin-based drugs are commonly used in chicken and pig rearing [6, 7]. The abuse of colistin in livestock may contribute to the introduction of CR bacteria. Supporting this hypothesis, Kawahara et al. [8] reported that in a rural area of Vietnam, approximately 100% chicken and pig fecal samples contained CR *Escherichia coli* with *mcr-1* or *mcr-3*. A retrospective study on extended-spectrum *β*-lactamase- (ESBL-) producing *E*. *coli* strains isolated from healthy residents during 2013–2016 in the Thai Binh Province of Vietnam showed that 6.9% of the ESBL-producing *E*. *coli* strains possessed *mcr-1* [9]. Another study from the same area conducted during 2017–2018 showed that approximately 70% of healthy residents carried CR *E*. *coli* with *mcr-1* and/or *mcr-3*, and that 92.8% of those isolates were multidrug resistant [10]. Thus, the extremely high prevalence of CR *E*. *coli* in healthy residents might be attributed to the transmission of these bacteria from livestock to humans via diet. Increased attention should be paid toward monitoring CR bacteria and CR-related genes in foods obtained from animal sources [11, 12].

As the presence of *E*. *coli* is indicative of food contamination, many studies have been conducted to detect the presence of CR *E*. *coli* in foods obtained from animals [13, 14]. However, these studies have only provided data on the rate of CR *E*. *coli* positivity and not on the abundance levels of CR *E*. *coli* in the samples. Here, we performed quantitative analysis of CR *E*. *coli* in chicken and pork meat samples for clarifying the status of the contamination of meat samples from local Vietnamese markets.

## 2. Materials and Methods

### 2.1. Sample Collection

Fifteen samples each of chicken and pork meat were purchased from 10 stores in two local markets in Thai Binh, Vietnam, from November 2018 to July 2019. For each meat type, only one sample was collected from a store. At some stores, multiple samples were collected at different times. The samples were placed in individual plastic bags and kept in a cooler box with ice until use. Bacterial isolates were cultured from the samples on the same day of collection.

### 2.2. Bacterial Culture

Twenty-five grams of each meat sample was placed in a stomacher bag containing 225 mL of buffered peptone water and hand-homogenized for 2 min. The resulting supernatant was serially diluted (10-fold) using sterile saline. Next, 100 *μ*L of each diluted sample was inoculated onto both CHROMagar ECC (ECC) and CHROMagar COL-APSE (COL-APSE) (CHROMagar, Paris, France). ECC allows for the detection of *E*. *coli*-like and other coliform bacteria. COL-APSE is a selective medium for CR Gram-negative bacteria, often used to detect CR *E*. *coli* in food samples [8, 15]. The number of *E*. *coli*-like colonies that could be distinguished based on colony color on both agar plates was determined after 24 h of incubation at 37°C.

### 2.3. Selection of CR *E*. *coli*-Like Colonies

One representative *E*. *coli*-like colony grown on COL-APSE from each sample was isolated. In some samples, six randomly selected *E*. *coli*-like colonies from one COL-APSE agar plate were also isolated. These isolates were further characterized for bacterial identification, susceptibility to colistin, colistin resistance genes, and homology between isolates.

### 2.4. Characterization of the Isolates

The isolates were identified using biochemical tests with triple sugar iron slants (Japan BD, Tokyo, Japan), motility–indole–lysine medium (Japan BD), and cellobiose–lactose–indole–*β*-d-glucuronidase medium (Kyokuto Pharmaceutical, Tokyo, Japan).

Colistin susceptibility and minimum inhibitory concentrations (MICs) of the isolates were determined using the standard broth microdilution method [16]. MICs ≥ 4 *μ*g/mL were interpreted as resistance to colistin.

The colistin resistance gene, *mcr*, was assessed using multiplex PCR. In brief, DNA was extracted from the isolates by boiling the bacterial suspension in tris(hydroxymethyl)aminomethane (10 mM)–ethylenediaminetetraacetic acid (1 mM) buffer pH 8.0 (Nippon Gene, Tokyo, Japan). The presence of *mcr-1*, *mcr-2*, *mcr-3*, *mcr-4*, and *mcr-5* was detected using PCR, as described previously [11].

The genetic relatedness of isolates was assessed using pulsed-field gel electrophoresis (PFGE). *Xba*I-digested genomic DNA of isolates embedded in agarose was analyzed using the CHEF-DR III System (Bio-Rad, Hercules, CA, USA) according to a reported method [17].

## 3. Results

### 3.1. Detection of CR *E*. *coli* in Meat Samples

The contamination of meat samples with CR *E*. *coli* was assessed by culturing meat homogenate samples on COL-APSE. Representative *E*. *coli*-like colonies from each sample were isolated and identified using biochemical methods. All the samples contained *E*. *coli*, and resistance to colistin was also confirmed by assessing the MICs of the isolates. As shown in Table 1, 40% and 66.7% of pork and chicken meat samples, respectively, contained CR *E*. *coli.*

### 3.2. Quantitative Contamination Level of Meat Samples with CR *E*. *coli*-Like Bacteria

**Figure 1:**
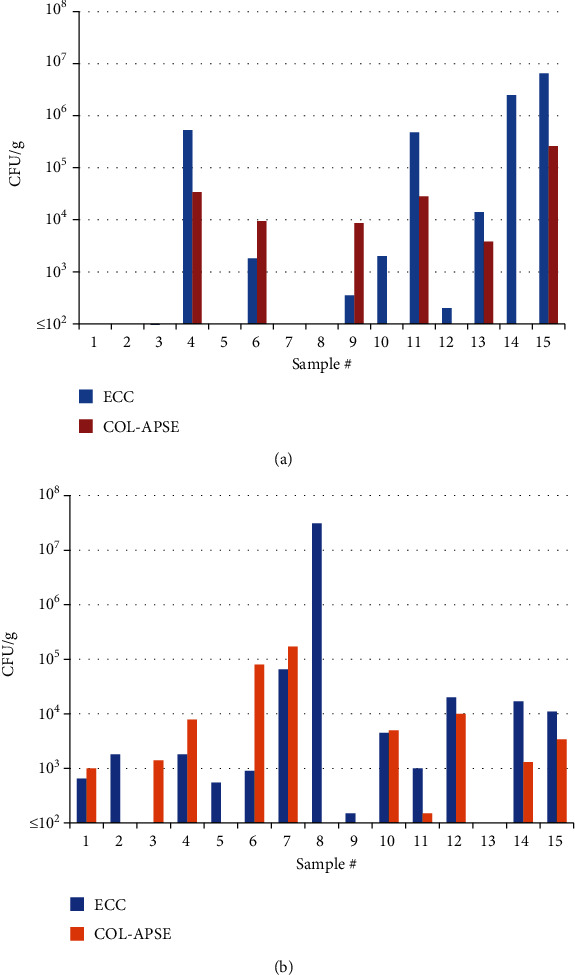
Levels of *Escherichia coli*-like and colistin-resistant *E*. *coli*-like bacteria from individual samples. (a) Pork meat samples; (b) chicken meat samples. ECC indicates the level of *E*. *coli*-like bacteria. COL-APSE indicates the level of colistin-resistant (CR) *E*. *coli*-like bacteria. See Materials and Methods for the quantification of bacteria in samples.

All sample homogenates were serially diluted and cultured on both ECC and COL-APSE for quantifying the levels of *E*. *coli*-like and CR *E*. *coli*-like bacteria. The levels of CR *E*. *coli*-like bacteria varied across samples (Figure 1). The mean levels in chicken and pork meat were 2.8 × 10^4^ and 5.7 × 10^4^ CFU/g, respectively, but the contamination frequency was lower in the latter (6/15 samples).

Because the number of *E. coli*-like colonies that grew on COL-APSE medium was high, none of the colonies were identified individually. Therefore, they were considered CR *E*. *coli*-like bacteria.

The level of *E*. *coli*-like bacteria in the samples was also determined by culturing the sample on ECC medium for comparing the CR bacteria levels. *E*. *coli*-like bacteria were detected in all CR-positive samples, except in chicken sample #3 (Figure 1(b)). Conversely, not all samples positive for *E*. *coli*-like bacteria showed the presence of CR *E*. *coli*-like bacteria, such as three samples of nine *E*. *coli*-like bacteria-positive pork samples and four samples of 13 *E*. *coli*-like bacteria-positive chicken samples were negative for CR-positive *E*. *coli*-like bacteria. The quantitative levels of *E*. *coli*-like bacteria also varied across samples (10^2^–10^7^ CFU/g).

### 3.3. Characterization of CR *E*. *coli* Isolates

**Figure 2:**
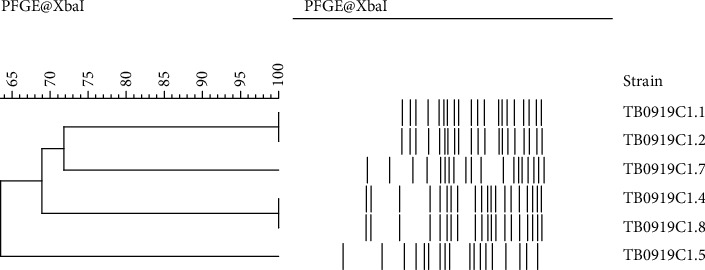
Dendrogram of pulsed-field gel patterns among colistin-resistant *Escherichia coli* colonies. A dendrogram of pulsed-field gel patterns among colistin-resistant *Escherichia coli* colonies on a CHROMagar COL-APSE agar plate cultured with a chicken meat sample homogenate was constructed. Strains from six randomly selected colonies from the plate were assessed.

The homology among various CR *E*. *coli* isolates derived from the same meat sample was assessed. Six randomly selected *E*. *coli*-like colonies grown on a COL-APSE plate by culturing a chicken sample were isolated, and colistin sensitivity and colistin resistance genes of these isolates were examined. The genetic relatedness of isolates confirmed to be CR *E*. *coli* possessing *mcr-1* was then assessed by PFGE analysis (Figure 2). The six isolates tested comprised four phylogenetically different clones, pulsotype 1 (TB0919C1.1 and TB0919C1.2), pulsotype 2 (TB0919C1.7), pulsotype 3 (TB0919C1.4 and TB0919C1.8), and pulsotype 4 (TB0919C1.5).

## 4. Discussion

After *mcr-1* was discovered in 2015, several retrospective studies showed that it is widely present in *E*. *coli* isolates from livestock, environment, food, and humans [2, 18–20]. However, the prevalence of *E*. *coli* possessing *mcr-1* varies widely in foods of animal origin, depending on the sample type and location and time of sampling. For instance, a study showed that the prevalence of *mcr-1-*harboring *E*. *coli* in chicken meat samples collected from The Netherlands between 2009 and 2014 was 1.5% [20], whereas another study on chicken meat samples collected from the same country in 2015 showed 24.8% prevalence [21]. Similarly, other studies have shown that the detection rate of *E*. *coli* possessing *mcr-1* from chicken meat samples collected from Germany [19], China [2], Japan [22], Brazil [23], and Nepal [24] ranged from 1.4% to 19.5%. More recently, a study in Tunisia showed that the rate of *E*. *coli* possessing *mcr-1* from a chicken meat sample was relatively high (38.3%) [25]. However, these detection rates were lower than those found in the present study (66.7%). Likewise, highly variable results have been reported for pork meat samples. The detection rates of *E*. *coli* possessing *mcr-1* in pork meat samples collected from China [2] and Japan [14] were 19.0% and 3.1%, respectively, whereas in Germany [19] and the Czech Republic [26], no pork meat sample showed contamination with *mcr-1*-harboring *E*. *coli*.

The detection rate of CR *E*. *coli* from pork and chicken meat samples in this study was higher than that in previous studies; however, the rate is consistent with that in our previous studies from the same area, which showed a high detection rate of *mcr*-harboring *E*. *coli* in fecal samples from healthy humans and livestock (human: 69.39%; chicken: 97.2%; pig: 94.4%) [8, 10]. The magnitude of such variations in the detection rate might largely be because of the sampling conditions mentioned above; moreover, the primary culture medium used for CR bacterial growth seems to affect the results.

As most studies till date have focused merely on the detection rate of *mcr*-positive CR *E*. *coli* in foods, the quantitative level remains unclear. Because a higher density of bacteria in food indicates a higher risk in terms of food safety, even the contamination density may be mainly superficial; the quantitative level of CR *E*. *coli* in food is practically an important indicator of food safety.

Our findings reveal high mean levels of contaminated CR *E*. *coli* in chicken and pork samples. To our knowledge, this is the first study on the quantitative analysis of the contamination of meat samples with CR *E*. *coli* possessing *mcr-1*.

As *E*. *coli* colonies were distinguished from other colonies only on the basis of color, we recognized these as *E*. *coli*-like bacteria in quantitative experiments. However, the representative colonies grown on COL-APSE were isolated from all samples and identified as CR *E*. *coli* using a standard biochemical identification method, susceptibility tests to colistin, and detection of *mcr* genes by PCR for the assessment of the contamination rate. The results showed that all these isolates were CR *E*. *coli* possessing *mcr-1*. Therefore, the results of the quantitative experiments were highly likely to indicate the level of CR *E*. *coli* in food samples, although not all colonies were identified in the experiments. Other *mcr* genes, such as *mcr-2* to *mcr-5*, were not detected in the CR *E*. *coli* isolates tested. The results showing *mcr-1* to be predominant are consistent with previous findings in livestock in the area [8].

Both ECC and COL-APSE can be used for quantifying the contamination levels in food samples. However, in some meat samples, the level of contamination with CR *E*. *coli*-like bacteria detected on COL-APSE was even higher than that with *E*. *coli*-like bacteria detected on ECC. This discrepancy might be caused by the presence of growth factor additives in COL-APSE, which is not disclosed by the manufacturer. Although it is not possible to determine the exact proportion of CR *E*. *coli* in the contaminating *E*. *coli*, with some exceptions, samples with a high number of contaminating *E*. *coli* might also have a high number of CR *E*. *coli* in food samples.

Generally, retail meats in local Vietnamese markets are processed as follows: shop owners in rural areas buy pigs and chickens, slaughter the animals themselves, and sell the meat before the day ends. The meat may get contaminated because of unhygienic slaughtering processes, the shopkeeper's hands, and flies or dirt present in the air during sale. Furthermore, because the meat is usually sold in hot and humid environments (average temperature during Vietnamese summers, 26–33°C; average humidity, >80%) without hygienic storage facilities, the bacteria can grow easily after contaminating the meat. Considering all these factors, clone population analysis of contaminated CR *E*. *coli* in food is important for understanding the actual situation. The results of PFGE analysis of CR *E*. *coli* isolates revealed that six randomly selected isolates from the same chicken sample belonged to four phylogenetically different clones, implying that these strains can contaminate foods from different sources during various stages of food processing, such as slaughtering, preservation, or sale. In contrast, four of the six *E*. *coli* isolates were classified into two genetically related clones, indicating that the bacteria may have multiplied after contaminating the food.

The results obtained in this study are not absolute because they easily varied depending on the situation of food, but they are important as an indication of the reality of CR *E*. *coli* contamination in the local community. Thus, the study shows that meat in local markets is frequently contaminated with certain levels of CR *E*. *coli*. Such highly contaminated meats may contribute to the dissemination of CR bacteria in the community.

## 5. Conclusions

The study assessed the quantitative levels of CR *E*. *coli* in meat samples from local Vietnamese markets. The results showed that pork and chicken meat were frequently contaminated with CR *E*. *coli* at levels of 10^4^ and 10^3^ CFU/g, respectively. Thus, the quantitative level of contamination with CR *E*. *coli* in retail meat has been clarified for the first time in this study.

## Figures and Tables

**Table 1:** Detection of colistin-resistant (CR) *Escherichia coli*-like bacteria in food samples.

	No. of samples tested	No. of samples contaminated with CR *E*. *coli*		Quantitative level of CR *E*. *coli*-like bacteria in samples (CFU/g)	
		#	%	Median	Range
Pork	15	6	40.0	1.8 × 10^4^	10^3^-10^5^
Chicken	15	10	66.7	4.2 × 10^3^	10^2^-10^5^

## Data Availability

The datasets used and/or analyzed during the current study are available from the corresponding author on reasonable request.

## References

[1] Kasiakou S. K., Michalopoulos A., Soteriades E. S., Samonis G., Sermaides G. J., Falagas M. E. (2005). Combination therapy with intravenous colistin for management of infections due to multidrug-resistant Gram-negative bacteria in patients without cystic fibrosis. *Antimicrobial Agents and Chemotherapy*.

[2] Liu Y. Y., Wang Y., Walsh T. R. (2016). Emergence of plasmid-mediated colistin resistance mechanism MCR-1 in animals and human beings in China: a microbiological and molecular biological study. *The Lancet Infectious Diseases*.

[3] Shen Y., Zhang R., Schwarz S. (2020). Farm animals and aquaculture: significant reservoirs of mobile colistin resistance genes. *Environmental Microbiology*.

[4] Catry B., Cavaleri M., Baptiste K. (2015). Use of colistin-containing products within the European Union and European economic area (EU/EEA): development of resistance in animals and possible impact on human and animal health. *International Journal of Antimicrobial Agents*.

[5] Nakayama T., Jinnai M., Kawahara R. (2017). Frequent use of colistin-based drug treatment to eliminate extended-spectrum beta-lactamase-producing *Escherichia coli* in backyard chicken farms in Thai Binh Province, Vietnam. *Tropical Animal Health and Production*.

[6] Van Cuong N., Nhung N. T., Nghia N. H. (2016). Antimicrobial consumption in medicated feeds in Vietnamese pig and poultry production. *EcoHealth*.

[7] Nguyen N. T., Nguyen H. M., Nguyen C. V. (2016). Use of colistin and other critical antimicrobials on pig and chicken farms in southern Vietnam and its association with resistance in commensal *Escherichia coli* bacteria. *Applied and Environmental Microbiology*.

[8] Kawahara R., Fujiya Y., Yamaguchi T. (2019). Most domestic livestock possess colistin-resistant commensal *Escherichia coli* harboring *mcr* in a rural community in Vietnam. *Antimicrobial Agents and Chemotherapy*.

[9] Kawahara R., Khong D. T., Le H. V. (2019). Prevalence of *mcr-1* among cefotaxime-resistant commensal *Escherichia coli* in residents of Vietnam. *Infection and Drug Resistance*.

[10] Yamamoto Y., Kawahara R., Fujiya Y. (2019). Wide dissemination of colistin-resistantEscherichia coliwith the mobile resistance genemcrin healthy residents in Vietnam. *Journal of Antimicrobial Chemotherapy*.

[11] Yamaguchi T., Kawahara R., Harada K. (2018). The presence of colistin resistance gene *mcr-1* and *-3* in ESBL producing *Escherichia coli* isolated from food in Ho Chi Minh City, Vietnam. *FEMS Microbiology Letters*.

[12] Perez-Rodriguez F., Mercanoglu Taban B. (2019). A state-of-art review on multi-drug resistant pathogens in foods of animal origin: risk factors and mitigation strategies. *Frontiers in Microbiology*.

[13] Ghafur A., Shankar C., GnanaSoundari P. (2019). Detection of chromosomal and plasmid-mediated mechanisms of colistin resistance in _Escherichia coli_ and _Klebsiella pneumoniae_ from Indian food samples. *Journal of Global Antimicrobial Resistance*.

[14] Nishino Y., Shimojima Y., Suzuki Y. (2017). Detection of the *mcr-1* gene in colistin-resistant *Escherichia coli* from retail meat in Japan. *Microbiology and Immunology*.

[15] Yamamoto Y., Calvopina M., Izurieta R. (2019). Colistin-resistant *Escherichia coli* with *mcr* genes in the livestock of rural small-scale farms in Ecuador. *BMC Research Notes*.

[16] Wikler M. A. (2006). Methods for dilution antimicrobial susceptibility tests for bacteria that grow aerobically: approved standard. *CLSI (NCCLS)*.

[17] Ribot E. M., Fair M. A., Gautom R. (2006). Standardization of pulsed-field gel electrophoresis protocols for the subtyping of *Escherichia coli O157:H7, Salmonella*, and *Shigella* for PulseNet. *Foodborne Pathogens and Disease*.

[18] Shen Z., Wang Y., Shen Y., Shen J., Wu C. (2016). Early emergence of *mcr-1* in *Escherichia coli* from food-producing animals. *The Lancet Infectious Diseases*.

[19] Irrgang A., Roschanski N., Tenhagen B. A. (2016). Prevalence of *mcr-1* in *E. coli* from livestock and food in Germany, 2010-2015. *PLoS One*.

[20] Kluytmans–van den Bergh M. F., Huizinga P., Bonten M. J. (2016). Presence of *mcr-1*-positive *Enterobacteriaceae* in retail chicken meat but not in humans in the Netherlands since 2009. *Euro Surveillance*.

[21] Schrauwen E. J. A., Huizinga P., van Spreuwel N., Verhulst C., Kluytmans-van den Bergh M. F. Q., Kluytmans J. (2017). High prevalence of the *mcr-1* gene in retail chicken meat in the Netherlands in 2015. *Antimicrobial Resistance and Infection Control*.

[22] Ohsaki Y., Hayashi W., Saito S. (2017). First detection of an *Escherichia coli* strain harboring the *mcr-1* gene in retail domestic chicken meat in Japan. *Japanese Journal of Infectious Diseases*.

[23] Monte D. F., Mem A., Fernandes M. R. (2017). Chicken meat as a reservoir of colistin-resistant *Escherichia coli* strains carrying *mcr-1* genes in South America. *Antimicrobial Agents and Chemotherapy*.

[24] Joshi P. R., Thummeepak R., Leungtongkam U. (2019). The emergence of colistin-resistant *Escherichia coli* in chicken meats in Nepal. *FEMS Microbiology Letters*.

[25] Hassen B., Abbassi M. S., Ruiz-Ripa L. (2020). High prevalence of *mcr* -1 encoding colistin resistance and first identification of *bla*_CTX-M-55_ in ESBL/CMY-2-producing *Escherichia coli* isolated from chicken faeces and retail meat in Tunisia. *International Journal of Food Microbiology*.

[26] Gelbicova T., Barakova A., Florianova M. (2019). Dissemination and comparison of genetic determinants of *mcr*-mediated colistin resistance in *Enterobacteriaceae* via retailed raw meat products. *Frontiers in Microbiology*.

